# Cancer, metabolism, fructose, artificial sweeteners, and going cold turkey on sugar

**DOI:** 10.1186/1741-7007-12-8

**Published:** 2013-01-31

**Authors:** Lewis C Cantley

**Affiliations:** 1 Department of Medicine, Weill Cornell Medical College, New York, NY 10065, USA

## 

Lewis Cantley graduated from West Virginia Wesleyan College in chemistry and took his PhD in biophysical chemistry at Cornell University where he worked on enzyme kinetics. He did his postdoctoral studies at Harvard University where he stayed as an assistant professor until he moved to Tufts University where he discovered phosphoinositide-3-kinase, the enzyme critical to the control of growth that has dominated his research ever since. He returned to Harvard as a Professor of Cell Biology and later as a member of the new Department of Systems Biology and is now Director of the new Cancer Center at Weill Cornell Medical College and New York-Presbyterian Hospital.

**Figure:**
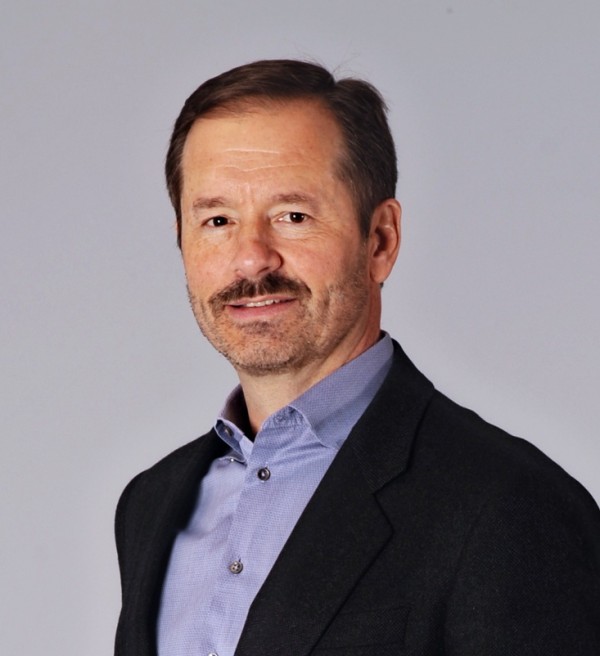
Lewis Cantley

## Quite early in your research career, you discovered the enzyme phosphoinositide kinase - usually known as PI3K - whose crucial activities are still central to your research. Can you briefly say what it does?

PI3K generates a lipid - and a lipid that wasn’t known prior to our discovery. It’s a very minor lipid - that’s why it had been missed - that is the product of inositol phosphorylation mediated by PI3K. Even at the time of our discovery that PI3K makes this novel lipid, we already had evidence that high levels of this enzyme correlated with malignant transformation of cells: in collaborations with Tom Roberts, Brian Schaffhausen and Ray Erickson we had shown that viruses that cause cancers in mice and chickens often do so by activating this novel enzymatic activity. So we knew early on that well-studied viral oncoproteins such as Src and polyoma middle T activate this lipid kinase. We went on to show that the product of PI3K, phosphatidylinositol-3,4,5-trisphosphate (PIP_3_), was quite high in cells transformed by these viruses. Because of its correlation with cancer, we suspected very early on that PIP_3_ was an oncolipid - although we didn’t actually name it that.

## So the connection with cancer was established very early on and then, in an overview of PI3K signaling that you wrote in 2002 [1], you predicted that studies on the PI3K pathway would lead to new targets for diabetes and cancer. Did you realize then that metabolic disturbances like diabetes and cancer might actually be linked?

Well, we did because we published a paper in 1990 where we reported that not only was PI3K activated by growth factors like EGF and PDGF, but it was also activated by insulin [2]. In fact, insulin turned out to be the very best way to activate it. As we continued to pursue that finding through the very early 1990s, we and others found that virtually everything that insulin did required the activity of PI3K. In other words, inhibitors of PI3K or knockouts of PI3K in mice abrogated insulin signaling. Insulin-dependent glucose uptake, for example, required PI3K.

And as we were doing our work in mammalian systems - on cell lines and mouse knock-outs - other labs were studying worms and flies, and the gene encoding PI3K popped up in genetic mutants of flies, in a pathway that was downstream of the insulin/insulin-like growth factor (IGF) receptor. While mammals have separate but related receptors for insulin and IGF1, flies and worms have a single receptor that is the ancestor of these two receptors. So PI3K showed up genetically in the insulin/IGF-1 signaling pathway that controls cell growth in flies. In worms, it popped up in a nutrient-dependent age-related phenotype. In fact, it was called 'age-1' before it was identified as PI3K because loss-of-function mutations in the gene dramatically extend the lifespan of worms. The genetic network for ageing turned out to be the insulin receptor, IRS-1, PI3K, AKT, FoxO network - the same network that we were uncovering in mammalian systems.

So it was very clear by the late 1990s that PI3K evolved as a mediator of insulin/IGF-1 receptor signaling. And as full genome sequences of flies and worms became available it became clear that while the insulin receptor-PI3K-AKT-FoxO pathway was well conserved, other pathways for activating PI3K that we had found in mammalian cells were less conserved. And that led us to conclude that PI3K and PIP_3_ originally evolved to mediate insulin/IGF-1 signaling and to control nutrient uptake - particularly glucose uptake in response to feeding - and distribute it into the appropriate tissues for the organism to grow.

## What you are mostly focused on now is specific disturbances of growth-related signaling networks in tumor cells that might suggest new drug targets, and I’d like to ask you about one noticeable thing about these studies - the frequent discovery of apparently paradoxical results. Would you like to say why these studies so often throw up paradoxes?

I think what we’re learning, of course, is that biological systems are far more complicated than we’d imagined. As we acquire tools that allow us to acutely knock out or knock down the expression of a particular gene, or have a drug that inhibits a particular step in a metabolic pathway or signaling pathway, we are finding that the system responds to these perturbations by attempting to reactivate the pathway. In other words, a lot of what we call robustness in nature comes about because biological systems have numerous negative feedback regulatory networks that sense when the system is out of balance and become altered to restore homeostasis. So some of the paradoxes come from the fact that whenever you inhibit a component of a signaling or metabolic network, you end up reactivating things upstream of it, giving a result that’s the opposite of what you expected to see.

In metabolic networks particularly, it’s been known for a long time that there are all kinds of feedback control. One example of a paradox from our research was the observation that pyruvate kinase, which is the enzyme in glycolysis that synthesizes ATP, actually tends to get turned down in cancer cells. This is paradoxical because cancer cells are typically utilizing glucose at 50- to 100-fold the rate of the normal tissue surrounding it, so why would they want to turn down one of the steps in that pathway and make less ATP? In the end, we figured out that it’s because that allows the cells to use the intermediates in glycolysis for other purposes than just making ATP, such as making NADPH, or making ribose, or making serine or glycine [3].

## These are for biosynthetic pathways?

That’s right. The cancer cell of course needs to grow, and it needs to be able to control its oxidation-reduction potential. Those are typically a greater challenge for a cancer cell than just making ATP, which it can do through oxidative phosphorylation in mitochondria. So if you turn down ATP synthesis through glycolysis because you’re using glucose intermediates for metabolic processes, you can make up for that in the mitochondria and the cell is fine.

## So would you conclude that you really need to know your way around metabolism before you can start to predict what will happen if you interfere with a particular step in the pathway?

That’s right. It’s only now that we have the tools to acutely perturb metabolic systems and monitor what happens, and we can really begin to understand the wiring diagrams of these pathways.

## I’d like to ask you one last question, on fructose. You’ve written recently on the very topical issue of whether fructose is a particularly important cause of metabolic disease [4] and, as we now know, with possible very strong links to cancer. As I understand it, sucrose - and even other carbohydrates - in excess can be metabolized to fructose. So if we’re just eating too much carbohydrate generally, does it really matter whether it’s fructose or any other kind?

It turns out that it does matter. Quite honestly, four or five years ago I was in your camp of assuming, you know - fructose, glucose, they have exactly the same number of calories per gram, they can be interconverted instantly inside most cells, so what does it matter? The answer is, it’s really important - and quite striking - because the liver differentially metabolizes fructose and glucose. This specialization is pretty much unique to the liver; in any other cell, the fructose and glucose are pretty much interchanged quite rapidly. But liver does not have hexokinase, so it cannot phosphorylate fructose at the six position. This is in contrast to glucose, which can be phosphorylated at the six position in the liver by glucokinase to make glucose-6-phosphate, which is then converted to fructose-6-phosphate. And that is then phosphorylated at the one position by phosphofructokinase (PFK), which is - and here’s the key point - the ultimate gatekeeper for entering glycolysis. In contrast, fructose that enters the liver is phosphorylated at the one position by fructokinase (also called ketohexokinase) to make fructose-1-phosphate rather than fructose-6-phosphate. The liver is almost unique in regard to the ability to differentially metabolize glucose and fructose.

## And that matters because…?

That matters because once it’s phosphorylated at the one position, fructose can be a substrate for aldolase, and shoot down the glycolytic pathway, bypassing the gatekeeper PFK, which is the control step for going into glycolysis. In most tissues, if the cell finds itself with plenty of ATP and plenty of citrate (the building blocks for making fatty acids), it will stop all flux through glycolysis because ATP and citrate inhibit PFK - a classic example of a metabolic negative feedback control. So the glucose that enters the cell can still get phosphorylated but it doesn’t go down glycolysis and doesn’t get converted to fat but rather gets stored as glycogen or exits the cell.

But in the liver, fructose bypasses that whole machinery, because it doesn’t need PFK; it gets phosphorylated at the one position directly, without phosphorylation of the six position first and, as a consequence, now becomes a substrate for aldolase, and it produces even higher levels of ATP and citrate that go on to make fatty acids. No matter how much you’ve eaten, you will still make more fat if you eat fructose.

There are two other things about fructose that make it different from glucose. One is that all the fructose you eat is cleared on its first pass through the liver. In other words, the liver scarfs up all the fructose and immediately converts it to fat, while glucose stays in the bloodstream for some period of time. That’s why we call starches hyperglycemic molecules; they keep glucose levels in your bloodstream high for a long time. That is good for the brain - the brain loves to eat glucose. It’s good for the muscle. But fructose doesn’t actually supply any energy to your brain at all, it doesn’t supply any energy to your muscle; it only gets stored as fat. That’s really quite remarkable, if you think about it. You eat sucrose - one molecule of glucose and one molecule of fructose - that glucose is being used by your muscle and your brain - your brain loves getting that glucose - but the fructose is all just getting stored as fat.

## But does it also mean that you get hungrier - you want more sugar if you’re using fructose rather than glucose?

Exactly. You would have to eat exactly twice as much sucrose as starch to get the same amount of energy supplied to your muscle and brain. The brain realizes that, it keeps relaying a feedback so that the more sugar you eat, the more it wants you to eat. Hence the addiction to sweetness. That’s the dangerous thing about this molecule.

You might ask - well why did we evolve such a complicated system? Why does only the liver feed fructose straight into fat? I think it’s quite clear why this happens. We have a symbiotic relationship with plants. Plants want to spread their seeds around, so they surround them with fructose. High-fructose material surrounding the seeds gets us and other animals to eat them and this craving of fructose makes us eat them a lot and we end up carrying their seeds around and spreading them. But at the same time, it gives us an advantage because those fruits ripen just at the end of the growing season, which generally means, in almost all environments, that you’re not going to have much to eat over the next few months. So the best way to survive is to convert everything you eat at that time into fat. That is the long-term storage mechanism that allows you to survive until the next growing season. That’s why fructose was spectacular for us 10,000 years ago, getting us through these famines that we faced every year. But today we don’t have famines and so we just get fat.

## Does this put a whole new gloss on Eve and that apple?

You’d probably have to eat about a bushel of apples to get the same amount of fructose as in a 40 oz Coke, which we’re trying to ban here in New York City unsuccessfully.

And here’s an additional comment. The way we’ve attempted to avoid this problem is by using artificial sweeteners. The problem with those is that a disconnect ultimately develops between the amount of sweetness the brain tastes and how much glucose ends up coming to the brain.

So the brain figures you have to eat more and more and more sweetness in order to get any calories out of it. The consequence of people eating lots of sweeteners, no matter what they are - whether they’re natural or unnatural - is that it increases the addiction for the sweetness. As a consequence, at the end of the day, your brain says, 'OK, at some point I need some glucose here'. And then you eat an entire cake, because nobody can hold out in the end. The only way really to prevent this problem - to break the addiction - is to go completely cold turkey and go off all sweeteners - artificial as well as fructose. Eventually the brain resets itself and you don’t crave it as much.
